# Chronic cadmium exposure decreases the dependency of MCF7 breast cancer cells on ERα

**DOI:** 10.1038/s41598-019-46912-3

**Published:** 2019-08-20

**Authors:** Mathew Bloomfield, Maggie C. Louie

**Affiliations:** Department of Natural Sciences and Mathematics, Dominican University of California, 50 Acacia Avenue, San Rafael, CA 94901 USA

**Keywords:** Breast cancer, Environmental sciences

## Abstract

Cadmium is an environmental contaminant that can activate estrogen receptor alpha (ERα) and contribute to the development and progression of breast cancer. Our lab previously demonstrated that chronic cadmium exposure alters the expression of several ERα-responsive genes and increases the malignancy of breast cancer cells. Although these studies support cadmium’s function as a hormone disrupter, the role of ERα in cadmium-induced breast cancer progression remains unclear. To address this, we modulated the expression of ERα and found that while the loss of ERα significantly impaired cancer cell growth, migration, invasion and anchorage-independent growth in both MCF7 and MCF7-Cd cells, cadmium-exposed cells retained a significant advantage in cell growth, migration, and invasion, and partially circumvented the loss of ERα. ERα knockout in MCF7 and MCF7-Cd cells significantly reduced the expression of classical ERα-regulated genes, while non-classical ERα-regulated genes were less impacted by the loss of ERα in MCF7-Cd cells. This is the first study to show that chronic cadmium exposure, even at low levels, can increase the malignancy of breast cancer cells by decreasing their dependency on ERα and increasing the adaptability of the cancer cells.

## Introduction

Breast cancer is the most common malignancy affecting women in the United States. Approximately 60–70% of breast cancers express estrogen receptor-alpha (ERα), and life-time exposure to estrogens, including those from the environment, is known to contribute to the development of breast cancer^1,2^. Cadmium, a metalloestrogen found ubiquitously in the environment, has been classified as a human carcinogen^3,4^. Exposure to low levels of cadmium— which bioaccumulates in tissues over time— occurs primarily through diet and cigarette smoke.

Epidemiological studies have linked cadmium exposure and ER-positive breast cancer^5–7^. A study comparing cadmium concentrations in tissue, blood, and urine of malignant and benign breast cancer patients showed that cadmium levels were significantly higher in patients with malignant tumors than those with benign tumors and that ERα-positive breast cancers had significantly higher cadmium concentrations than ERα-negative cancers^5^. These correlations suggest that cadmium might be a critical factor in tumors expressing ERα. Additional epidemiological studies found that cadmium increases breast cancer risk, tumor malignancy, and metastasis frequency^5,8,9^. In one study, ovariectomized animals exposed to cadmium exhibited increased uterine weights and high densities of epithelial cells in the mammary gland, but these effects were not observed in animals concurrently treated with the antiestrogen ICI-182,780 (ICI), suggesting that ERα may play an important role in mediating cadmium’s physiological effects^10^. Similarly, Alonso-Gonzalez *et al*. showed an increase in uterine weight, ductal branching, and lobuloalveolar development in ovariectomized mice after 7 weeks of exposure to cadmium^11^. Accordingly, *in vitro* studies have indicated that cadmium has estrogenic activity^12–15^. Cadmium activated ERα at concentrations as low as 10^−11^ M and blocked estradiol binding in a noncompetitive manner, indicating that cadmium interacts with ERα in the ligand binding domain^12^. Our lab found that MCF7 cells exposed to low levels of cadmium for six months had a unique gene expression profile and increased growth, migration, and invasion capabilities, indicating that chronic cadmium exposure promotes breast cancer progression^16,17^ by altering the interactions among ERα, c-jun, and c-fos^16^ and promoting the expression of SDF1, a chemokine regulated by ERα^18,19^.

Despite evidence that cadmium acts as a metalloestrogen and can promote breast cancer progression, it is unclear whether the estrogenic activity of cadmium is critical for cancer progression, especially under chronic low-level exposure^20,21^. A study by Benbrahim-Tallaa *et al*. demonstrated that prolonged exposure to cadmium malignantly transformed breast epithelial cells *in vitro*, independent of ERα expression, indicating that the estrogenic effects of cadmium are not required for transformation^20^. The objective of this study was to determine the role of ERα in cadmium-mediated breast cancer progression. Our results demonstrate that although ERα plays an important role in cadmium-induced gene expression and mediates malignant phenotypes, chronic cadmium exposure also decreases the dependency of MCF7 cells on ERα.

## Results

Exposure to cadmium has been associated with increased breast cancer risk and malignancy^5,8^. Although there is evidence suggesting that cadmium functions as a metalloestrogen, it is unclear whether this mechanism directly contributes to the development and progression of breast cancer. To determine the role of ERα in cadmium-induced breast cancer progression, we used the CRISPR/Cas9 gene editing system to permanently knock out ERα expression in parental MCF7 cells and two previously established cadmium-adapted MCF7 clonal cell lines (Cd7 and Cd12)^16^. DNA sequencing and protein expression analysis of the MCF7, Cd7, and Cd12 CRISPR-edited clones revealed that 8 contained DNA sequence mutations that resulted in a loss of ERα protein expression (Supplementary Fig. S1) and were therefore selected for further characterization. Clones that continued to express ERα of either the same or different molecular weights were not used for further analysis.

**Figure 1:**
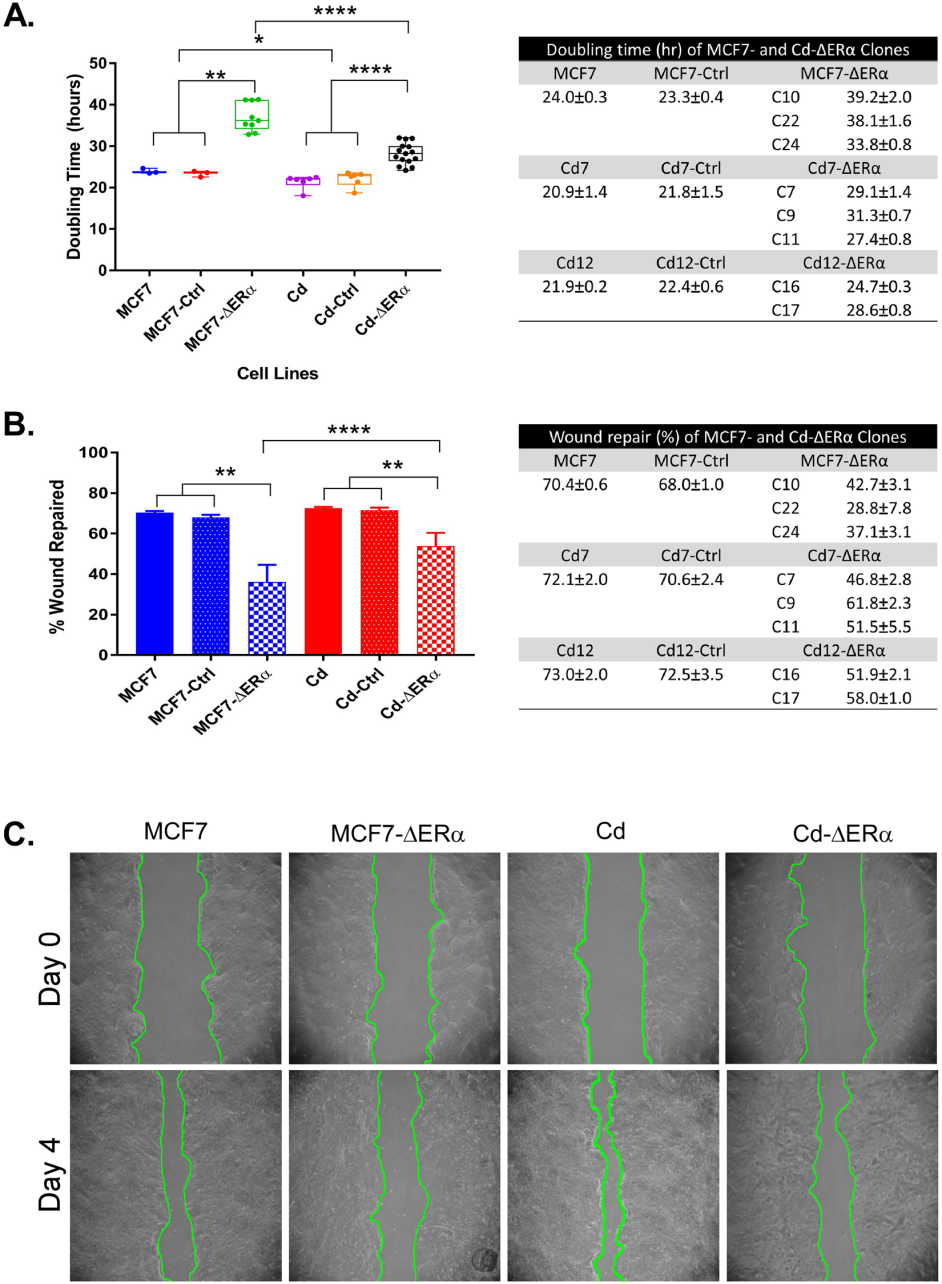
Characterization of MCF7, Cd7, and Cd12 cells after ERα knockout. (**A**) Approximately 5 × 10^4^ cells were seeded in 6-well plates, and the total cell number was determined after 24, 72, 96, and 120 hours to calculate the population doubling time of each cell line. MCF7-ΔERα included MCF7-C10, MCF7-C22, and MCF7-C24 clones, and Cd-ΔERα included Cd7-C7, Cd7-C9, Cd7-C11, Cd12-C16, and Cd12-C17. The data were derived from the means of three independent experiments (with standard error of mean (SEM); *n* = 3, 3, 9, 6, 6, 15, left to right) and analyzed using the Wilcoxon-Mann-Whitney test to determine statistical significance (*p < 0.05; **p < 0.01; ****p < 0.0001). Table shows the average doubling times of each cell line (*n* = 3 for all). (**B**) Migration ability for each control and clone was quantified by comparing the surface area of the scratch wounds at day 0 and day 4. The data represent the means of three independent experiments of triplicate samples with SEM (*n* = 3, 3, 9, 6, 6, 15, left to right). Data analysis was performed using the Wilcoxon-Mann-Whitney test (**p < 0.01; ****p < 0.0001). Table shows the average migration ability of each cell line (*n* = 3 for all). (**C**) Representative images of MCF7, MCF7-ΔERα, Cd (MCF7-Cd), and Cd-ΔERα cells at days 0 and 4 with the wound outlined in green.

To investigate how the loss of ERα affects the phenotypes of MCF7 and cadmium-adapted cells (MCF7-Cd), we measured the doubling times for all the clones lacking ERα (ΔERα) compared to those of the control cells by determining the total cell number at day 0, 2, 3, and 4. For statistical analysis, three MCF7-ΔERα clones (C10, C22, and C24) served as biological replicates (n = 3), while the three Cd7-ΔERα (C7, C9, and C11) and two Cd12-ΔERα (C16 and 17) clones were biological replicates of cadmium-adapted, ERα knockout cells (n = 5). Consistent with previous data^16^, the results in Fig. 1A show that the cadmium-adapted cells grew faster than the MCF7 cells (24.0 vs 21.4 hours; p < 0.05). No significant differences in growth were observed between the MCF7 and MCF7-Ctrl or MCF7-Cd and MCF7-Cd-Ctrl groups, indicating that transfection with control plasmids had little phenotypic impact (Fig. 1A). As expected, the loss of ERα in MCF7-ΔERα increased the doubling time from 24 hours (MCF7) to an average of approximately 37 hours (p < 0.01; Fig. 1A). In the case of the cadmium-adapted cells, the loss of ERα in Cd-ΔERα cells increased the doubling time from 21.4 hours (MCF7-Cd) to 28.2 hours (p < 0.0001; Fig. 1A). Therefore, despite the loss of ERα, Cd-ΔERα clones retained a significant growth advantage over the MCF7-ΔERα cells (28.2 vs 37.0 hours; p < 0.0001), which was even more significant than the difference between the MCF7-Cd and MCF7 cells (Fig. 1A).

To determine whether depletion of ERα affects the ability of cadmium-adapted cells to migrate, we used a scratch wound assay. In brief, cells were grown to 80–90% confluence, a scratch wound was inflicted to the monolayer, and the migratory ability of the cells to repair the wound was monitored over 4 days. The difference in surface area of the wound from day 0 to day 4 was calculated using ImageJ software. The results in Fig. 1B,C show that both the wounds in the MCF7 and MCF7-Cd cells were almost fully closed by day 4, while this ability was reduced in Cd-ΔERα cells and even more significantly impaired in MCF7-ΔERα cells. More specifically, the loss of ERα decreased the migratory ability from 70.4% (MCF7) to an average of 36.2% (MCF7-ΔERα; p < 0.01), while the loss of ERα in MCF7-Cd cells reduced this ability from 72.6% to an average of 54% (Cd-ΔERα; p < 0.01; Fig. 1B,C). The migratory ability of the Cd-ΔERα clones was significantly greater than that of the MCF7-ΔERα clones (p < 0.0001; Fig. 1B). Similar to cell growth, ERα is important for migration, although in the absence of ERα, the cadmium-adapted cells still exhibit a high migratory potential.

**Figure 2:**
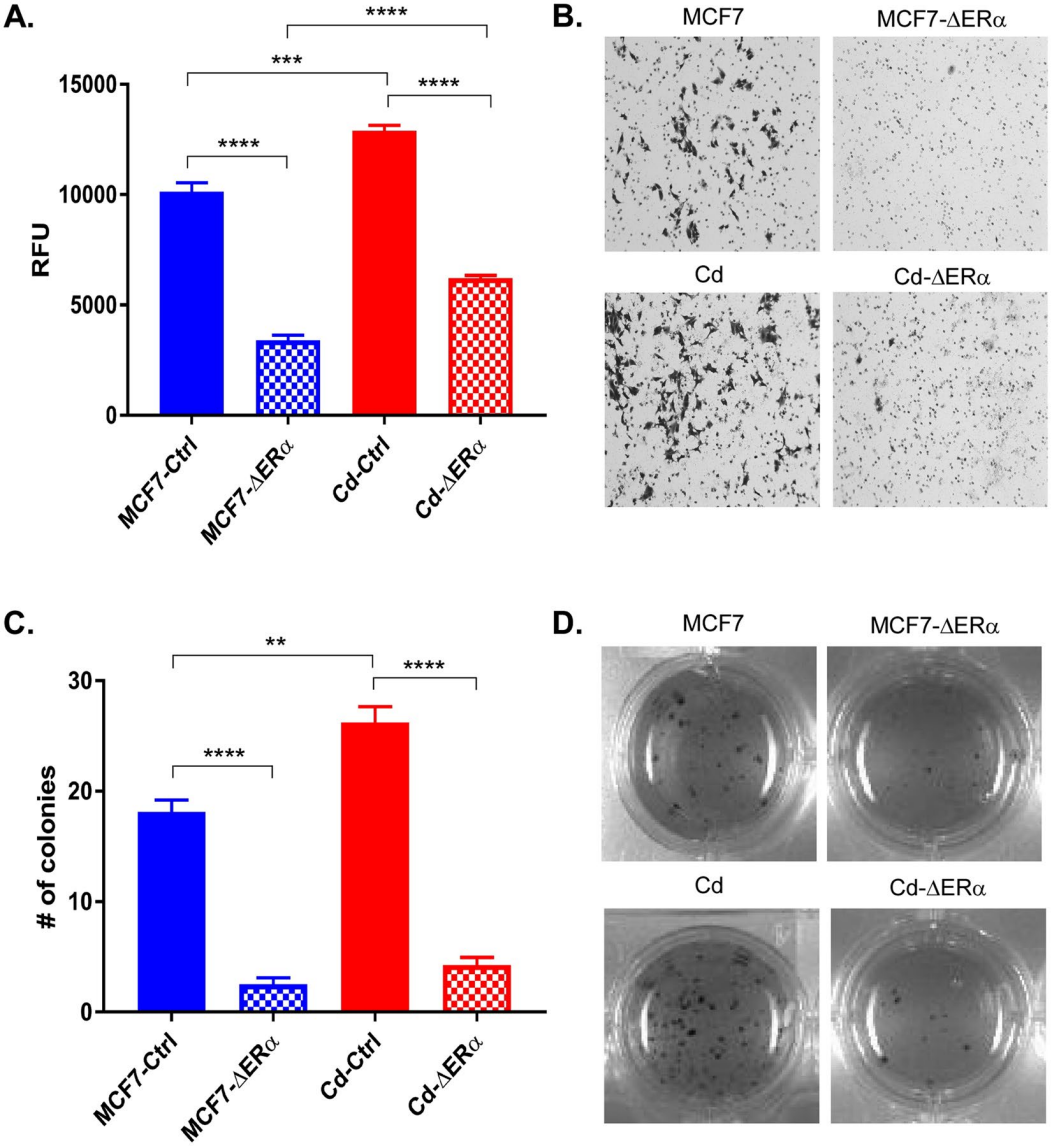
Cadmium-adapted cells are more invasive and tumorigenic than parental MCF7 cells. (**A**) Approximately 5 × 10^4^ cells were seeded into cell invasion chambers in triplicate and incubated for 24 hours. Invasive cells were measured using a fluorescent plate reader, and the data was collected from three independent experiments and shown as mean with SEM (*n* = 9, 27, 18, 45, left to right). Statistical analysis was performed using a two-tailed T test (***p < 0.001; ****p < 0.0001). (**B**) Representative images of invasive MCF7-Ctrl, MCF7-ΔERα, Cd-Ctrl (MCF7-Cd), and Cd-ΔERα cells stained with crystal violet. The results are representative of three independent experiments. (**C**) Anchorage-independent growth was measured using the colony formation assay in soft agar. Only colonies with approximately 100 cells or more were counted, and the results represent three independent experiments of triplicate samples shown as mean with SEM (*n* = 9, 27, 18, 45, left to right). Statistical analysis was performed using a two-tailed T test (**p < 0.01; ****p < 0.0001). (**D**) Representative images of MCF7, MCF7-ΔERα, Cd, and Cd-ΔERα colonies growing in soft agar.

Given the differences observed in both growth and migration between MCF7-ΔERα and Cd-ΔERα clones, the invasive ability of these cells was measured. Cells were seeded in the upper level of a modified Boyden chamber and incubated for 24 hours. Cells that successfully invaded the membrane were either quantified by measuring fluorescence (Fig. 2A) or compared after staining with crystal violet (Fig. 2B). Consistent with our prior observations^16^, the MCF7-Cd cells were more invasive than the MCF7 cells (p < 0.001; Fig. 2A,B). Similar to the results of the growth and migration analyses, the loss of ERα in both MCF7-ΔERα and Cd-ΔERα cells significantly reduced the invasive ability compared to that of their respective controls (p < 0.0001; Fig. 2A,B). Specifically, the invasiveness of the MCF7-ΔERα clones decreased 0.34-folds compared to parental MCF7 cells, while the Cd-ΔERα clones only decreased 0.51-folds relative to their control (Supplementary Fig. S2A). Thus, despite the loss of ERα, Cd-ΔERα cells continued to exhibit more invasive characteristics than MCF7-ΔERα clones (p < 0.0001; Fig. 2A,B).

As a final assessment of tumorigenic potential, we analyzed anchorage-independent growth using a soft-agar colony formation assay. Cells were seeded in soft agar and allowed to grow for two weeks, and colonies of 100-plus cells were counted (Fig. 2C,D). Consistent with the previous phenotypic analyses, MCF7-Cd cells formed significantly more colonies than the MCF7 cells (p < 0.01; Fig. 2C,D). The loss of ERα significantly reduced the number of anchorage-independent colonies formed from an average of 19 (MCF7) to approximately 2.5 in the MCF7-ΔERα cells (p < 0.0001; Fig. 2C,D). In cells chronically exposed to cadmium, MCF7-Cd cells formed an average of 27 colonies, whereas Cd-ΔERα only formed approximately 4.3 colonies (p < 0.0001). The difference in the number of colonies formed by Cd-ΔERα and MCF7-ΔERα cells was not statistically significant, although it was trending towards significance with a p-value of 0.064 (Fig. 2C). Additionally, when normalized to their respective controls, the fold changes of the MCF7-ΔERα and Cd-ΔERα clones were similar (Supplementary Fig. S2B). Collectively, the phenotypic analyses—growth, migration, invasion, and anchorage independence—confirmed that chronic cadmium exposure increases the tumorigenic potential of breast cancer cells and demonstrated the importance of ERα for these cancer characteristics. However, despite the loss of ERα, the cadmium-adapted cells retained growth, migration, and invasion advantages over MCF7 cells, suggesting that chronic cadmium exposure decreases the impact of ERα loss on breast cancer cells and enables cells to better adapt to the loss of ERα.

**Figure 3:**
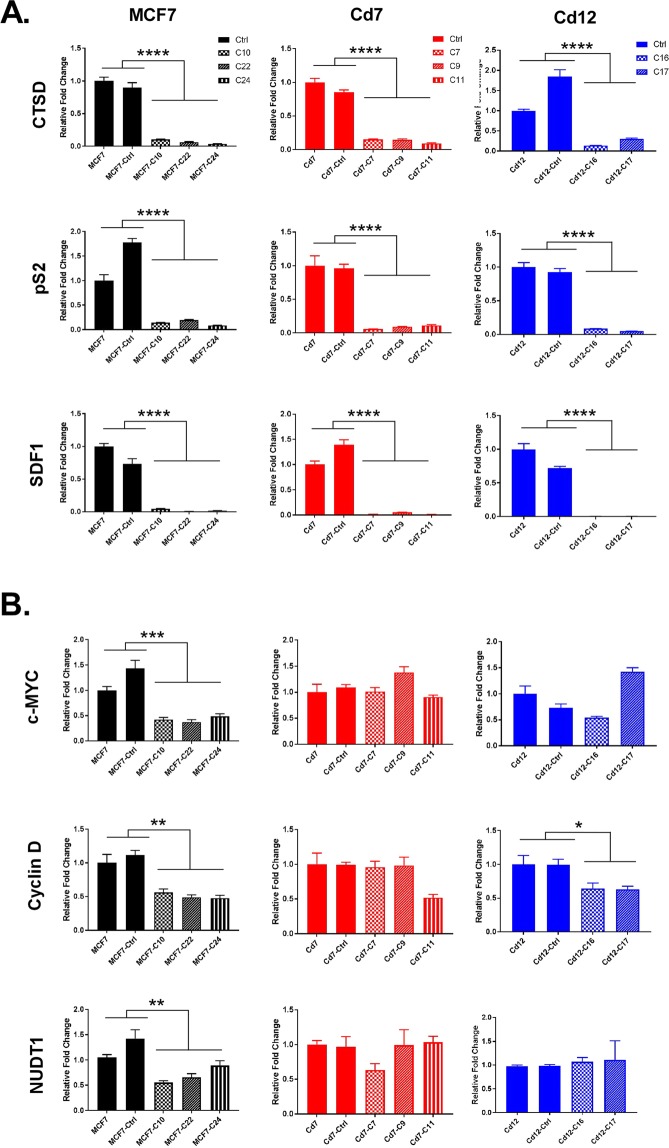
The effect of ERα knockout on the expression of classical and non-classical ERα genes. (**A**) Gene expression of the classical ERα genes CTSD, pS2, and SDF1 and the (**B**) non-classical genes, c-myc and cyclin-D1, and the estrogen-responsive gene NUDT1 were measured using RT-qPCR. MCF7-, Cd7-, and Cd12-Ctrl indicate the cells transfected with a CRISPR control plasmid. MCF7 was used as the control and all relative fold changes were normalized to actin (relative fold = 2^ΔΔCt gene/ΔΔCtActin^). The results represent the average of three independent experiments of quadruplicate samples (*p < 0.05; **p < 0.01; ***p < 0.001; ****p < 0.0001).

To further understand mechanistically how the loss of ERα in cadmium-adapted cells affects the expression of ERα-regulated and ERα-responsive genes, we analyzed the expression of three classical ERα-regulated (ERE) genes (CTSD, pS2, and SDF1) and three non-classically ERα-regulated or estrogen-responsive genes (c-myc, cyclin D1, NUDT1)^22,23^ using qRT-PCR analyses. The loss of ERα resulted in a significant reduction in CTSD, pS2, and SDF1 at the mRNA level in all cells—MCF7, Cd7, and Cd12 (Fig. 3A, p < 0.0001). The genes c-myc and NUDT1 were significantly downregulated in the MCF7-ΔERα cells (p < 0.01), while there were no significant decreases in the Cd7-ΔERα and Cd12-ΔERα cells (Fig. 3B). Interestingly, the loss of ERα had varying effects on cyclin D1 expression, with the most significant decrease in MCF7-ΔERα cells (p < 0.01), followed by Cd12-ΔERα cells (p < 0.05) and finally the Cd7-ΔERα cells, which showed no significant reduction (Fig. 3B). These findings demonstrate that ERα is critical for the expression of the classical ERE genes in all three cell lines; however, the cadmium-adapted cells appear to have an increased ability to continue expressing some non-classically ERα-regulated and estrogen-responsive genes despite the permanent loss of ERα.

To understand how chronic cadmium exposure alters the cells’ dependency on ERα for gene expression, we transiently silenced ERα using ICI, an antiestrogen that promotes the degradation of ERα^24–26^. Using a chemical inhibitor to directly reduce ERα levels allows analysis of the pathways altered immediately following this decrease, whereas the CRISPR ERα-KO cell lines would have already adapted to the ERα loss and changes may not reflect the immediate response.

**Figure 4:**
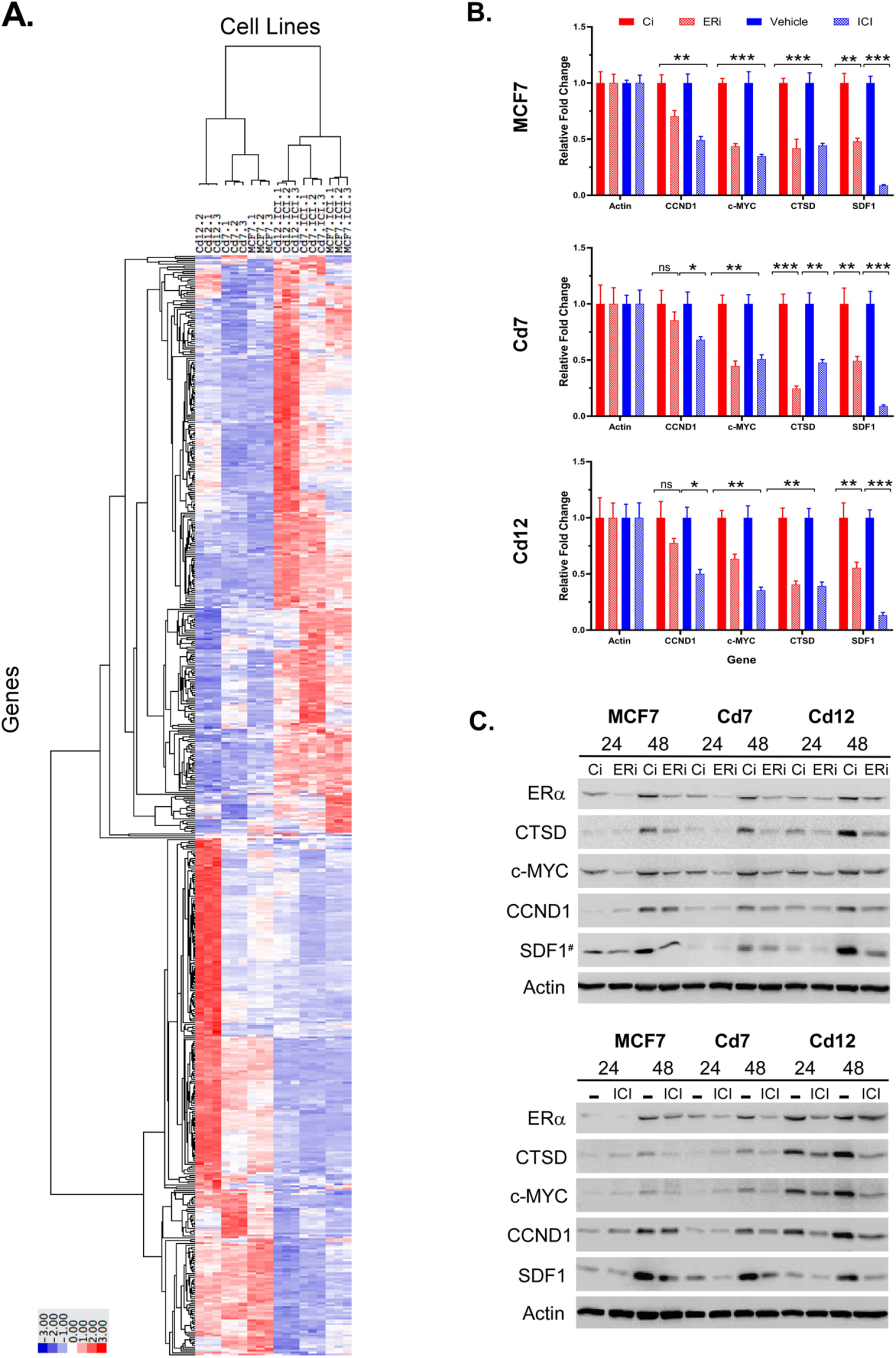
The effects of chronic cadmium exposure on ERα-responsive gene expression. (**A**) MCF7, Cd7, and Cd12 cells were treated with either the antiestrogen ICI-182,780 or vehicle in triplicate for 24 hours. Total RNA was collected, and RNA-seq was performed by the University of Minnesota Genomics Center. The top 500 differentially expressed genes (FDR ≤ 10^−6^) after ERα knockdown were hierarchically clustered. (**B**) MCF7, Cd7, and Cd12 cells were transfected with si-ERα (ERi) or si-control (Ci) or treated with 100 nM ICI-182,780 (ICI) or vehicle (−) and collected after 24 hours for gene expression analysis using RT-qPCR (*p < 0.05; **p < 0.01; ***p < 0.001). (**C**) Cell lysates were collected from MCF7, Cd7, and Cd12 cells 24 or 48 hours after ERα knockdown for protein expression analysis by western blot with actin as the loading control.

MCF7 and cadmium-adapted cells (Cd7 and Cd12) were treated with ICI to mediate the degradation of ERα, and a nonbiased global gene expression analysis was conducted using RNA sequencing (RNA-seq). The hierarchical clustering of the top 500 differentially expressed genes (false discovery rate (FDR) ≤ 10^−6^) in Fig. 4A shows that many ERα-regulated genes (i.e.GREB1, PR, SDF1, CTSD, NRIP1, IGF1R, and PRSS23)^12,16,17,27^ were upregulated in the Cd-adapted cells compared to the MCF7 cells, which supports our previous findings that the metalloestrogenic function of cadmium alters the expression of ER-regulated genes^16,17^. Strikingly, the RNA-seq analysis also showed that the loss of ERα even transiently resulted in global gene expression changes in both the MCF7 and cadmium-adapted cells (Fig. 4A). To confirm this data, we independently treated MCF7 and cadmium-adapted cells with ICI or RNAi to silence ERα (Fig. 4B,C) and subsequently analyzed the ERα target genes—SDF1, CTSD, c-myc, and cyclin D1 (CCND1)—using qRT-PCR and western blot analyses. Consistent with the RNA-seq data, depletion of ERα by either ICI or RNAi-ERα decreased the expression of the ERα target genes at both the transcript and protein levels (p < 0.05; Fig. 4B,C, Fig. S3).

**Figure 5:**
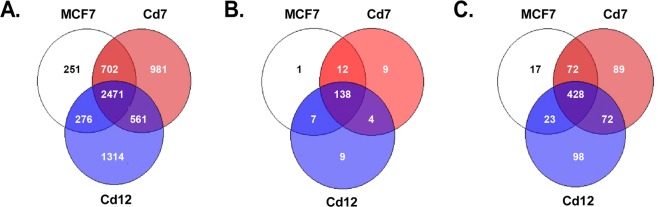
Prolonged cadmium exposure alters the regulation of estrogen-responsive genes. The Venn diagrams represent the (**A**) total, (**B**) ERE (estrogen response element), and (**C**) estrogen-responsive genes identified by RNA-seq that were altered in the same or different directions (up- or downregulated) in MCF7, Cd7, and Cd12 cells after antiestrogen treatment.

Subsequently, we performed pairwise comparisons of MCF7 vs. MCF7-ICI, Cd7 vs. Cd7-ICI, and Cd12 vs. Cd12-ICI and found that ICI-mediated degradation of ERα resulted in 3,706, 4,721, and 4,628 DE genes in MCF7, Cd7, and Cd12 cells, respectively. Of the DE genes, 2,477 were shared by all three cell lines (Fig. 5A). Overall, MCF7 shared 67.3% and 59.5% of the DE genes with Cd7 and Cd12 cells, respectively, suggesting that ERα continues to play an important role in regulating the expression of genes following chronic cadmium exposure. To gain insight into the biological functions and processes affected by ERα knockdown, Gene Ontology (GO) enrichment analysis was performed on the top 1,500 DE genes ranked by FDR in the MCF7 and cadmium-adapted cells after ICI treatment. Consistent with the similarities amongst the differentially expressed genes (Fig. 5A), many of the GO terms for molecular function (MF) and biological process (BP) were similarly enriched in all three cell lines following ICI treatment (Supplementary Fig. S4). As expected, modulating ERα expression altered common GO molecular functions such as “signaling receptor activity,” “transmembrane signaling receptor activity,” “molecular transducer activity,” and “catalytic activity,” in which ERα-regulated genes like CCND1, CTSD, and IGFR1 were present.

To further investigate how chronic cadmium exposure may impact ERα gene regulation, we compared the effects of ERα loss on the expression of ERE genes and estrogen-responsive genes^28,29^ specifically. The results in Fig. 5B show that 180 ERE genes were altered when ERα levels decreased. Of those, 138 ERE genes (76.7%) were shared by all three cell lines, in that expression changed in the same direction (either up- or downregulated) (Fig. 5B, Table S2A). For the estrogen-responsive genes, 428 (53.6%) of the 799 genes were altered in the same direction in all three cell lines (Fig. 5C, Table S2B). These findings show that while a majority of ERE genes responded in the same manner to loss of ERα, more variability existed within the estrogen-responsive genes. Collectively, these results indicate that while chronic cadmium exposure leads to genome-wide transcriptional changes, ERα remains important for regulating the expression of genes and maintaining the malignant phenotypes associated with breast cancer progression.

## Discussion

Epidemiological studies have reported a link between cadmium and breast cancer risk and malignancy^5,8,9^. Animal models have also shown that cadmium promotes early signs of cancer development in the mammary gland and uterus^10,11,30^. Although multiple *in vitro* studies have shown that acute levels of cadmium can mimic the effects of estrogen and activate ERα to alter the expression of target genes^13–16^, less is known about the effects of chronic, low-level cadmium exposure. Here, we investigated the effects of prolonged cadmium exposure on breast cancer progression and gene expression and the role of ERα in these processes. Our results demonstrated that cells chronically exposed to cadmium (MCF7-Cd) outperformed the parental MCF7 cells in the growth, invasion, and colony formation assays (Figs 1 and 2), extending previous observations that chronic cadmium exposure results in more aggressive cancer phenotypes^16,31–34^. The migration results of this study showed differences between MCF7 and MCF7-Cd cells (Fig. 2B,C), though the results were not as statistically significant as previous reported^16^. This may be because a pooled population of cadmium-adapted cells were used in the previous study rather than clonal-derived cell lines used here. In this current study, the loss of ERα significantly reduced the growth, migration, invasion and colony formation abilities in both the MCF7 and MCF7-Cd cells (Figs 1 and 2); however, this decrease was less pronounced in the cadmium cells, suggesting that cells chronically exposed to cadmium have become less dependent on ERα and perhaps have developed an increased ability to adapt to stresses—such as ERα loss.

To understand the molecular changes underlying these phenotypic differences, we also analyzed changes in gene expression after knocking out ERα using CRISPR/Cas-9. Knockout of ERα in both MCF7 and MCF7-Cd cells significantly reduced the levels of ERE genes, while non-classical ERα-regulated and estrogen-responsive genes, such as c-myc, cyclin-D1, and NUDT1, were less affected by ERα loss in the MCF7-Cd cells compared to MCF7 cells (Fig. 3). This may explain the enhanced aggression of the Cd-ΔERα cells as these genes are associated with cancer growth and invasiveness^35–37^. To capture the immediate response to the loss of ERα at the gene level, we used the antiestrogen ICI to transiently reduce ERα levels, and an unbiased gene expression analysis was performed using RNA-seq. Consistent with our ERα knockout results and our earlier observations that chronic cadmium exposure alters expression of ERα-regulated genes [e.g., PRSS23, CTSD, and SDF1^17^], transient loss of ERα also decreased the expression of many ERα target genes (Fig. 4A). Interestingly, c-myc and cyclin-D1 were downregulated in MCF7, Cd7, and Cd12 cells after transient silencing of ERα (Fig. 4B), while they were less affected in the cadmium-adapted cells after ERα knockout (Fig. 3B). This difference may be attributed to either incomplete loss of ERα under transient conditions or the fact that transient reduction of the receptor does not allow cells to adapt to the change. Nevertheless, the ability of the cadmium-adapted cells to recover the expression of these genes after ERα loss underscores the cells’ ability to adapt in comparison to parental MCF7 cells.

Consistent with the ERα knockout experiments, the RNA-seq analysis also revealed more variability in how estrogen-responsive genes were affected by the decreased ERα levels, with only 53.6% of the estrogen-responsive genes altered in the same direction in all three cell lines (MCF7, Cd7, Cd12) compared to 76.7% in the ERE genes (Fig. 5B,C). We speculate that prolonged cadmium exposure may have altered and expanded the function of ERα. Since cadmium is known to displace other divalent metals, such as zinc^38–40^, cadmium-bound ERα could have altered functions (i.e., transcriptional activity) and interactions with other proteins involved in transcription, as was previously observed in acute cadmium exposure^11,15,40^. Of course, it is possible that the differences in gene expression are not dependent on direct interactions between cadmium and ERα. Many estrogen-responsive genes are co-regulated by other transcription factors (e.g., AP-1, Sp-1) in partnership with ERα^41,42^, and alterations in the expression and/or activity of these transcription factors in the cadmium-adapted cells could also explain the differential response of estrogen-responsive genes to loss of ERα. However, it is unlikely that these observed differences in Cd7 and Cd12 cells are due to ERα-independent or off-target effects of ICI since (1) these genes have previously been shown to be estrogen-responsive, and (2) the ICI treatment conditions were the same in each cell line. However, how the effects of chronic cadmium exposure on activity of other steroid hormone receptors —ERβ, GR, and PR—remains unclear. Future studies to understand whether these transcriptional changes are mediated by changes in the cross-talk of these receptors and other transcription factors to ERα may offer further insights into how cadmium contributes to breast cancer progression.

Although our findings demonstrated that ERα remains critical for the development and maintenance of cadmium-induced malignant phenotypes in MCF7 cells, breast cancer cells chronically exposed to cadmium have developed additional mechanisms to partially circumvent the loss of ERα and continue to thrive. Consistent with these results, Benbrahim-Tallaa *et al*. demonstrated that the estrogenic effects of cadmium were not necessary for carcinogenesis after cadmium-mediated malignant transformation of MCF10A cells, an immortalized normal breast epithelial cell line that does not express ERα^20^. In line with previous observations^43–47^, our study does not dispute that cadmium induces changes independent of ERα, but also suggests that when present, ERα plays a critical role in cadmium-induced breast cancer progression. Collectively, our findings demonstrate for the first time that chronic cadmium exposure, even at low levels, can increase the malignancy of breast cancer cells by ultimately decreasing their dependency on ERα and thus increasing their adaptability.

## Materials and Methods

### Materials

MCF7 cells were obtained from the American Type Culture Collection (ATCC Manassas, VA). Cadmium chloride (Acros Organics, Geel, Belgium) was dissolved in autoclaved H_2_O and sterile-filtered to make a 1 M solution. A stock solution of ICI-182,780 (Tocris Bioscience, Bristol, UK) was prepared at a concentration of 10^−3^ M in DMSO according to the manufacturer’s protocol.

### Cell culture

MCF7 cells were obtained from the American Type Culture Collection (Manassas, VA) and cadmium-adapted cells (MCF7-Cd7 and MCF7-Cd12) were generated as described previously^16,17^. All MCF7 and derivative cell lines were maintained in Dulbecco’s modified Eagle’s medium (DMEM) (Life Technologies, Carlsbad, CA) supplemented with 10% fetal bovine serum (FBS) (HyClone, Logan, UT) and 1% penicillin and streptomycin (P/S) (Life Technologies). The media used for the cadmium-adapted cell lines MCF7-Cd7 and MCF7-Cd12 also contained 10^−7^ M CdCl_2_.

### Modulating ERα expression

#### RNA interference

Approximately 1 × 10^5^ cells were seeded into 6-well plates and transfected the following day with ERα siRNA (Santa Cruz Biotechnology, Santa Cruz, CA) using siRNA transfection reagents (Santa Cruz Biotechnology). A scrambled siRNA (Santa Cruz Biotechnology) was used as a control. The following day, the medium was replaced with DMEM containing 10% FBS and 1% P/S. Cells were harvested 24 and 48 hours later for gene and protein expression analysis using qRT-PCR and western blot analysis, respectively.

#### Derivation of CRISPR/Cas-9-edited cell lines

Approximately 1 × 10^5^ cells were seeded into 6-well plates and transfected with ERα double nickase plasmids (Santa Cruz Biotechnology). For selection of successfully transfected cells, 2.5 μg/mL puromycin (Santa Cruz Biotechnology) was added to the media for three days. Single cell clones were isolated by serial dilution in a 96-well plate, and wells with only a single cell were expanded into clonal cell lines. Cell lines were initially screened for ERα protein expression by western blot analysis using an ERα-specific antibody (Ab-12, Neomarkers). Clones that did not express ERα at the protein level were candidates for DNA sequencing verification performed by Genewiz, Inc. (South Planfield, NJ). Sequence reads of ~800 bp spanning the target region in the first exon of ESR1 were aligned using MacVector software (MacVector, Inc., Version 12.7.0 (214), Apex, NC) to identify frameshift mutations.

### Cell growth assay

Approximately 50,000 cells were plated in 6-well plates. The next day, cells from triplicate wells were counted twice using a hemocytometer to calculate total cell number (Thermo Fisher), and the total cell number was determined again 2, 3, and 4 days later after the initial cell count. The doubling times were determined using the exponential growth equation in GraphPad Prism v7.02 (GraphPad Software, Inc., San Diego, CA).

### Scratch wound assay

Cells were grown to approximately 80–90% confluence in 6-well plates before being scratched with a P200 pipette tip. The wound was imaged at Day-0 and again at Day-4. Cells were grown in supplemented DMEM media (see “*Cell Culture*” above) for the duration of the experiment. For quantification of cell migration, the surface area of the wound at days 0 and 4 was calculated using ImageJ (NIH, Bethesda, MD). The percent of the wound healed was calculated using the following equation:

$$ \% \,{\rm{wound}}\,{\rm{repaired}}=[1-({\rm{wound}}\,{\rm{surface}}\,{\rm{area}}\,{\rm{day}}\,4/{\rm{wound}}\,{\rm{surface}}\,{\rm{area}}\,{\rm{day}}\,0)]\times 100$$

### Invasion assay

Approximately 50,000 cells were seeded into ECMatrix cell invasion chambers (Millipore, Milpitas, CA) in triplicate and incubated for 24 hours per manufacturer’s protocol. Serum-free medium and DMEM with 10% FBS was added to top and bottom chambers, respectively. Luminescence was measured using a Fluorstar Omega plate reader (BMG Labtech, Ortenberg, Germany). For the images, approximately 50,000 cells were seeded in Corning^TM^ Biocoat^TM^ Matrigel^TM^ Invasion chambers in triplicate, and again, medium with no FBS was added to the top chamber, and medium with 10% FBS was added to the bottom chamber. After incubation for 24 hours, the cells on the bottom of the membrane were fixed in 10% PBS-buffered formalin for 30 minutes and then stained with crystal violet. The cells inside the chamber were removed, and the cells that invaded to the underside of the membrane were imaged using Motic Images Plus 2.0 (Motic, Richmond, British Columbia). Images are representative of 3–5 frames.

### Soft-Agar colony formation assay

Twenty-four-well plates were coated with 1 mL 1% agar in supplemented DMEM with 10% FBS and 1% P/S, and this constituted the bottom layer of the well. Approximately 500 cells were mixed with 0.5 mL 0.6% agar DMEM containing 10% FBS and 1% P/S, poured on top of the bottom layer in triplicate wells and incubated at 37°C and 5% CO_2_. Fresh medium was added every 2–3 days. After two weeks, live colonies were stained using MTT and imaged using the ChemiDoc Imaging system (Bio-Rad). Colonies greater than 100 cells were counted in triplicate wells.

### Protein expression analysis

Cells were lysed in 1% sodium dodecyl sulfate (SDS)-HEPES buffer (0.05 M HEPES, 1% Triton, 0.002 M EDTA, 1% deoxycholate, 0.002 M EGTA, 0.15 M NaCl, and 0.01 M NaF) plus protease inhibitor cocktail (Thermo Fisher, Waltham, MA) for 15 minutes at 4 °C. The cell lysate was then centrifuged at 20,000 × g for 15 minutes at 4 °C. The total protein concentration was determined using the Bio-Rad DC Protein Assay kit (Bio-Rad, Inc., Hercules, CA). Proteins were separated using SDS-polyacrylamide gel electrophoresis and transferred to polyvinylidene fluoride (PVDF) membranes (Millipore, Hayward, CA). The membranes were blocked with 5% milk-Tris-buffered saline with Tween-20 (TBST) for one hour before protein expression was monitored using the following specific antibodies at dilutions ranging from 1:500 to 1:1000: ERα Ab-12 (6F11) (Neomarkers, Fremont, CA), Cathepsin D (C-5; Santa Cruz), SDF1 (Cell Signaling Technology), c-myc (D84C12; Cell Signaling Technology), Cyclin D (A-12; Santa Cruz), and Actin (AC-15; Sigma). HRP-goat anti-mouse and -rabbit secondary antibodies (Invitrogen, Carlsbad, CA) were used at a concentration of 1:2000, and Clarity Western ECL Substrate (Bio-Rad) was used for detection. Images were captured and analyzed using the iBright CL1000 imager (Invitrogen). Antibodies used to recognize specific proteins were highly specific and have been previous studies^15,16^. A representative full length blot of each target is found in Supplemental Fig. S2.

### Quantitative reverse transcription polymerase chain reaction (qRT-PCR)

Total RNA was isolated from cells using TRIzol reagent (Life Technologies) and columns from the Direct-zol RNA MiniPrep kit (Zymo Research Corporation, Irvine, CA) according to the manufacturer’s protocol. One microgram of total RNA was converted to cDNA using the High Capacity RNA-to-cDNA kit (Applied Biosystems, Inc., Foster City, CA). Gene expression was quantified using gene-specific primers (Table 1) and Fast SYBR Green master mix (Applied Biosystems). The reaction was cycled 40 times with an annealing temperature of 60ºC. All gene-specific primers were synthesized by Integrated DNA Technologies, Inc. (IDT, San Diego, CA).

**Table 1:** List of qRT-PCR Primer Sequences.

	qRT-PCR Primer Sequences (5′ → 3′)
c-myc_F_ c-myc_R_	CTCCACACATCAGCACAACT GTTTCCGCAACAAGTCCTCT
cyclin D1_F_ cyclin D1_R_	AATGTGTGCAGAAGGAGGTC GAGGGCGGATTGGAAATGAA
CTSD_F_ CTSD_R_	CTCTGTCCTACCTGAATGT GACAGCTTGTAGCCTTTG
SDF1_F_ SDF1_R_	GTCAGCCTGAGCTACAGATGC CACTTTAGCTTCGGGTCAATG
pS2_F_ pS2_R_	GCGCCCTGGTCCTGGTGTCCA GAAAACCACAATTCTGTCTTTC
NUDT1_F_ NUDT1_R_	ATGGACGTGCATGTCTTC TGTGTAGTCCAGGATGGT
Actin_F_ Actin_R_	GAGAAAATCTGGCACCACACC ATACCCCTCGTCGATGGGCAC

### Identifying differentially expressed (DE) genes

Cells were treated with 10^−7^ M of the antiestrogen ICI or vehicle, and total RNA was isolated as described above. Triplicate samples were sent to the bioinformatics core at the University of Minnesota Genomics Center (Minneapolis, MN). RNA was sequenced using a HiSeq-2500 (Illumina, Hayward, CA) to produce 50-bp paired-end reads at a depth of 22,000X. EdgeR^48^ was used to determine DE genes, and the resulting list of genes was ranked by the false discovery rate (FDR), which ranged from 10^−3^ to 10^−6^. Different subsets of data were compared using Perl scripts (www.perl.org). Cluster 3.0^49,50^ was used to organize data sets by DE genes, and heatmaps highlighting the top 500 genes were created using http://jtreeview.sourceforge.net/. The complete RNA-seq data sets are available at the NCBI’s Gene Expression Omnibus (GEO) (accession GSE134127).

### Functional enrichment of DE genes

The top 1,500 DE genes (ranked by FDR) identified from pairwise comparisons of MCF7 vs. MCF7-ICI, Cd7 vs. Cd7-ICI, and Cd12 vs. Cd12-ICI were used as input for Gene Ontology (GO) enrichment analysis (geneontology.org)^51^. PANTHER GO-Slim Molecular Function and Biological Process enrichment (p < 0.05) was determined using Fisher’s Exact test.

### Statistical analysis

Normality was determined using the Shapiro-Wilk test in GraphPad Prism v7.02. Data following a normal distribution were analyzed using a two-tailed T test in GraphPad Prism to determine the statistical significance as specified in figure legends. A non-parametric T test was used where the *n* value was too small to determine normality.

## Supplementary information

Supplemental figures and legends

Table S1A

Table S1B

Table S2A

Table S2B
