# Supplementary material for: Chronic cadmium exposure decreases the dependency of MCF7 breast cancer cells on ERα
Source: Sci Rep. 2019 Aug 20;9:12135. doi: 10.1038/s41598-019-46912-3 (PMC6702212; doi:10.1038/s41598-019-46912-3)
Supplement: Supplementary file 1 — Supplemental figures and legends [file 41598_2019_46912_MOESM1_ESM.pdf]

TITLE: Chronic cadmium exposure decreases the dependency of MCF7 breast cancer cells on ERα

AUTHORS: Mathew Bloomfield[1] and *Maggie C. Louie[1]

[1]Department of Natural Sciences and Mathematics; Dominican University of California; 50 Acacia Avenue, San Rafael, CA 94901

*Corresponding author
Phone: (415) 485-3248
Fax: (415) 482-1972
Email: maggie.louie@dominican.edu

*Reprint requests should be addressed to the corresponding author.

EMAIL ADDRESSES
MCL: maggie.louie@dominican.edu
MB: mbloomfield@vt.edu

KEYWORDS: Breast cancer, cadmium, chronic exposure, estrogen receptor, ERα

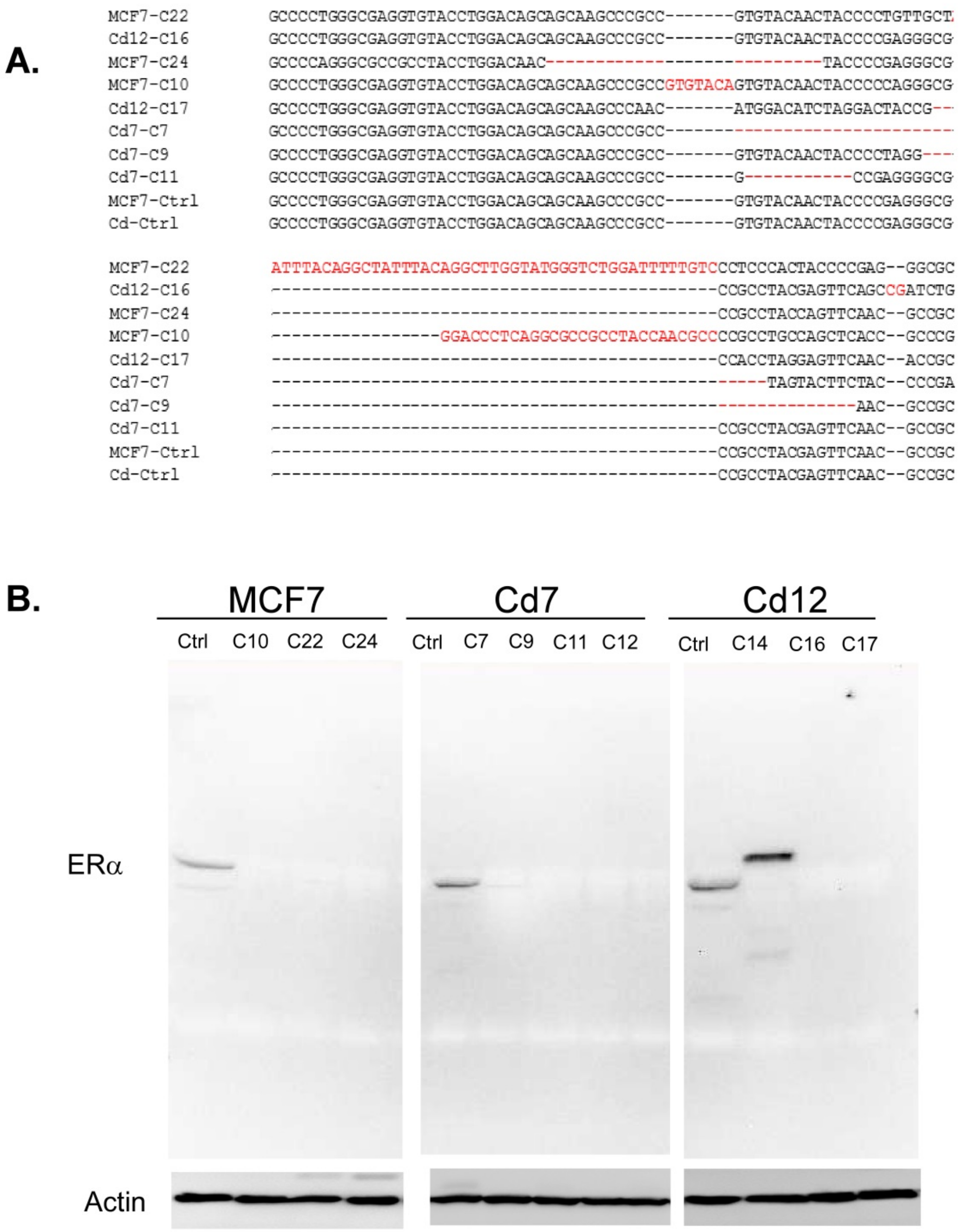

Supplementary Figure S1. Generation of ERα-KO clones using the CRISPR/Cas9 gene-editing system. (A) DNA sequence alignment showing a region that flanks the CRISPR target sequence showing the presence of insertion or deletion mutations (shown in red letters) in single cell-derived clones after transfection with plasmids containing sgRNA oligos and Cas9 enzyme. (B) The presence or absence of ERα in control or CRISPR-transfected clones was verified using western blot analysis with actin as the loading control.

A.

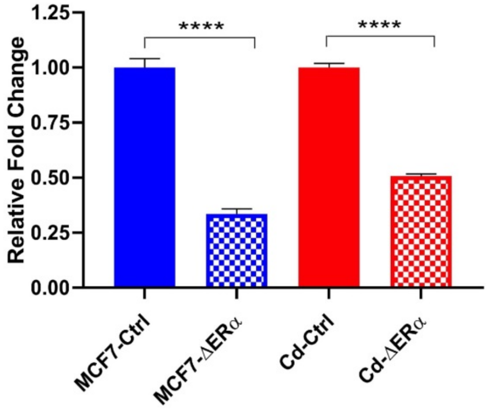

B.

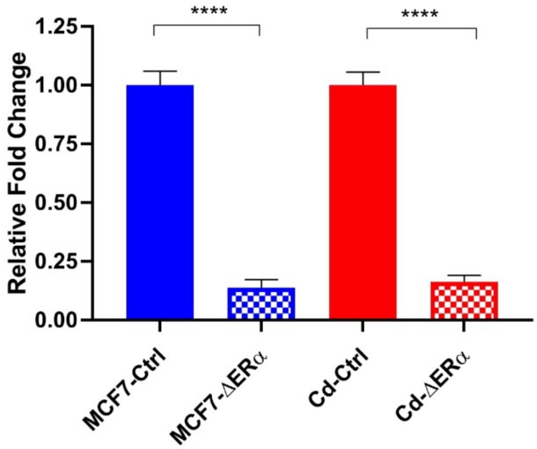

Supplementary Figure S2. Normalized invasion and colony formation abilities of MCF7-ΔERα and Cd-ΔERα clones. Bar graphs of the invasiveness (A) and tumorigenicity (B) of the MCF7-ΔERα and Cd-ΔERα clones shown as relative fold change to normalize for baseline differences existing between MCF7 and Cd cells (****$p<0.0001$).

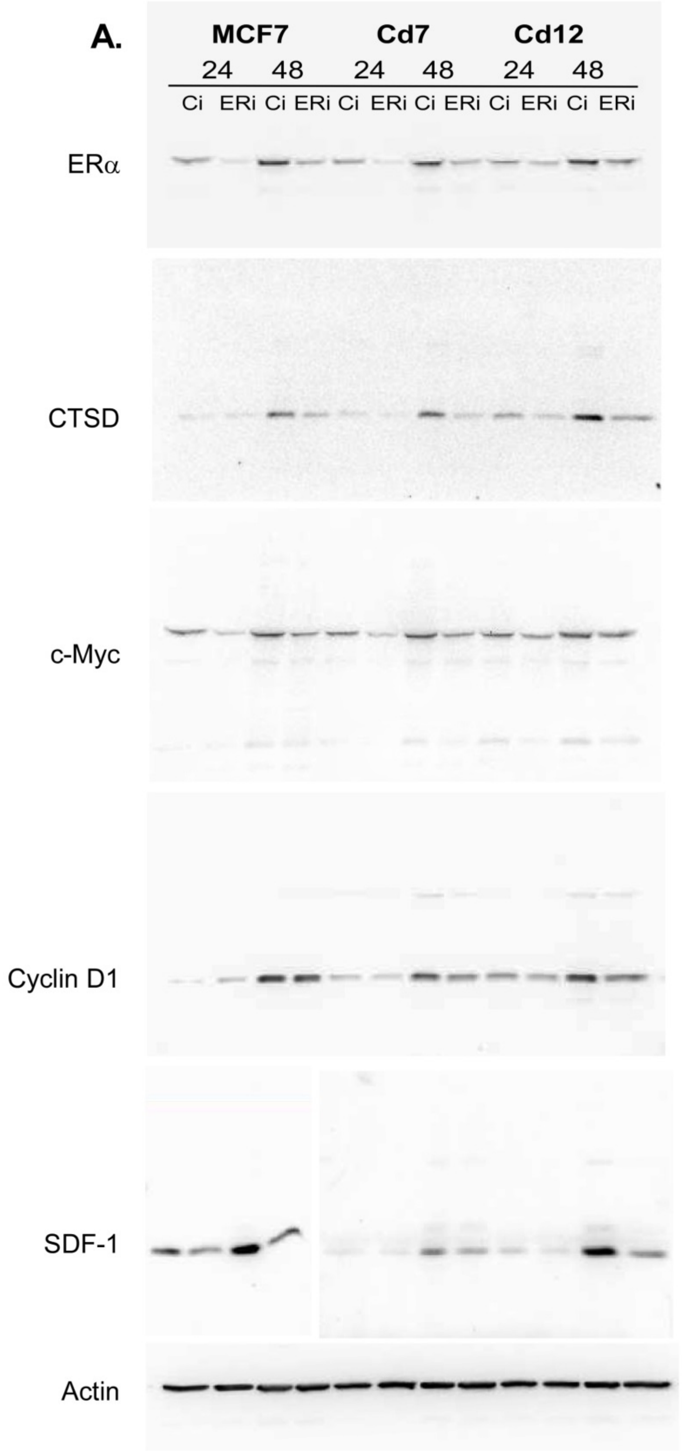

Supplementary Figure S3. Full-length of Western Blot Images for Figure 4C.
MCF7, Cd7, and Cd12 cells were transfected with si-ERα (ERi) or si-control (Ci) and cell lysates were collected from MCF7, Cd7, and Cd12 cells 24 or 48 hours after ERα knockdown for protein expression analysis using western blots with actin as the loading control. #SDF-1 blot is composed of two blots— one with MCF7 cells and the other with Cd7 and Cd12 cells, each transfected with either si-ERα or si-control.

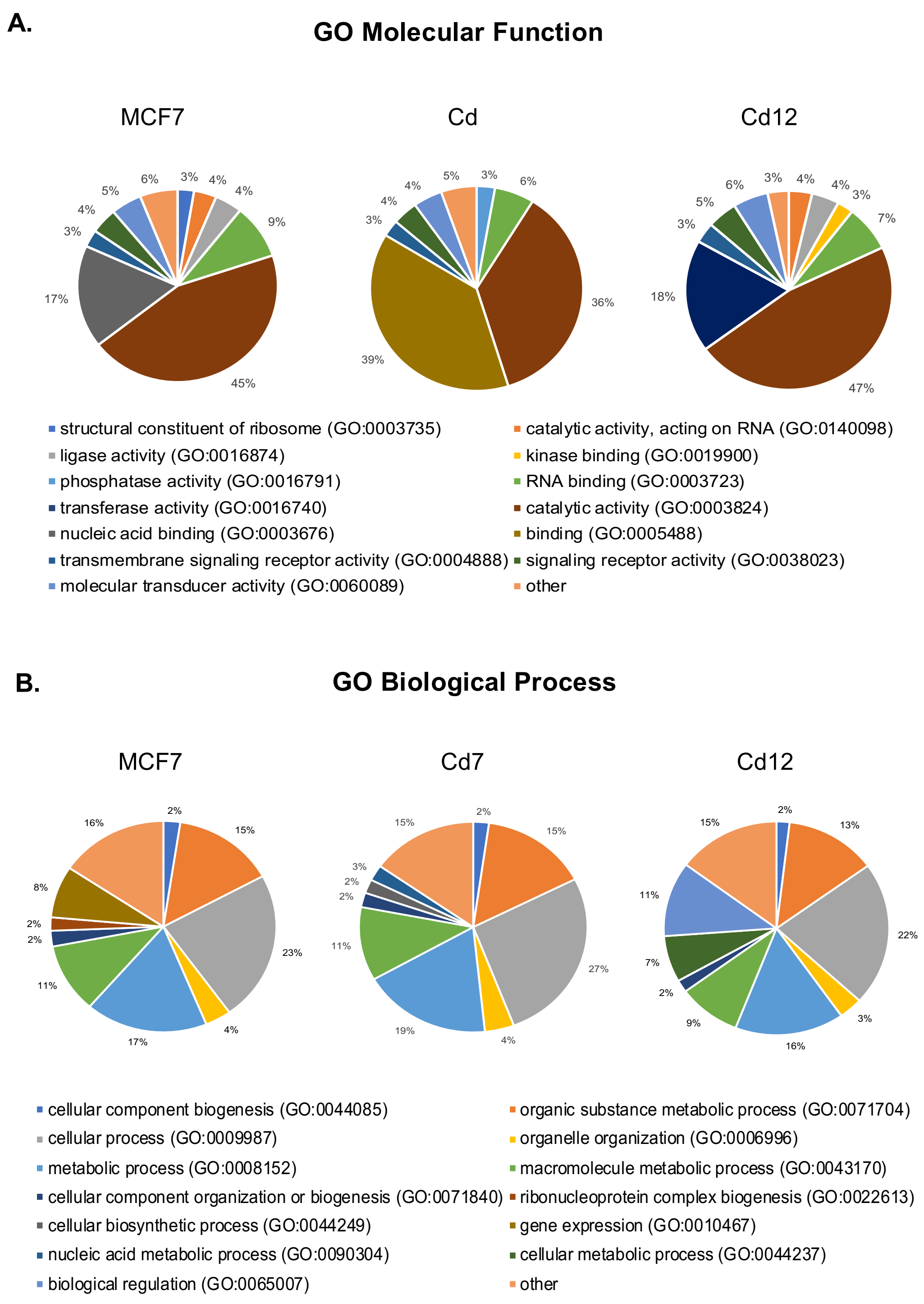

Suplementary Figure S4. Enrichment of GO terms in MCF7, Cd7, and Cd12 cells after ERα knockdown. Pie charts showing the over-represented (A) Molecular Function and (B) Biological Process using PANTHER GO-Slim in parental and cadmium-adapted MCF7 cells following exposure to ICI. Annotations where the corresponding number of DE genes was less than 1% were categorized as “other.”
